# Case Report: Pediatric CNS-isolated hemophagocytic lymphohistiocytosis secondary to uniparental disomy of *PRF1* mutation

**DOI:** 10.3389/fgene.2025.1528844

**Published:** 2025-07-21

**Authors:** Jiao Xue, Zhenfeng Song, Hongshan Zhao, Chengqing Yang, Fei Li, Zhi Yi, Kaixuan Liu, Ying Zhang

**Affiliations:** ^1^ Department of Pediatric Neurology, The Affiliated Hospital of Qingdao University, Qingdao, Shandong, China; ^2^ Department of Anesthesiology, The Affiliated Hospital of Qingdao University, Qingdao, Shandong, China

**Keywords:** central nervous system, hemophagocytic lympho-histiocytosis, PRF1 gene, uniparental disomy, pediatric

## Abstract

**Background:**

Central nervous system-isolated hemophagocytic lymphohistiocytosis (CNS-HLH) is a rare disease caused by mutations in several genes.

**Methods:**

Clinical information was obtained from medical records. Genetic analyses were performed using whole-exome sequencing (WES). NK cell function testing, Granzyme B staining, perforin staining, CD107a mobilization, and soluble CD25 levels were determined.

**Results:**

We report the case of a 5-year-old girl who presented with involuntary movements, an unsteady gait, and a progressively irritable temper. Cranial MRI revealed bilateral multifocal white matter abnormalities. The patient harbored a homozygous missense mutation in the *PRF1* gene (NM_001083116.3), c.1349C > T (p.Thr450Met), which is a maternal uniparental disomy. Based on the phenotype and absence of perforin expression, the patient was diagnosed with CNS-HLH.

**Conclusion:**

We report a highly unusual case of CNS-HLH diagnosed by uniparental disomy of a *PRF1* mutation. Exome sequencing should be considered in patients with chronic or recurrent brain inflammation who show partial or no response to conventional treatment regimens.

## 1 Introduction

Hemophagocytic lymphohistiocytosis (HLH) is a multisystem inflammatory disorder that results from an unregulated cytokine storm and activation of cytotoxic T cells and antigen-presenting cells (Gupta et al., 2023). HLH typically affects multiple organ systems. HLH rarely involves the central nervous system, even in the absence of systemic inflammatory features (Benson et al., 2019). Here, we report a highly unusual case of a 5-year-old girl who presented with involuntary movements and an unsteady gait, and was diagnosed with central nervous system isolated HLH (CNS-HLH) by harboring uniparental disomy of a *PRF1* mutation (c.1349C > T, p. Thr450Met).

## 2 Methods

The patient was admitted to our hospital in October 2024. Clinical information was obtained from medical records. Genetic analysis was performed using whole-exome sequencing (WES). NK cell functional testing, Granzyme B staining, perforin staining, CD107a mobilization, and soluble CD25 levels were determined at the Beijing Hightrust Diagnostic Medical Laboratory. Neural antibodies associated with autoimmune encephalitis, including NMDAR, LGI1, CASPR2, GABA_B_R, AMPAR1, AMPAR2, IgLON5, DPPX, GAD65, mGluR5, GlyR, D2R, MOG, and GFAP in serum and cerebrospinal fluid (CSF) were tested using Cell-Based Assays with immunofluorescence double staining at the Jiangsu Simcere Diagnostic Laboratory (Jiangsu Simcere Diagnostics Co, Ltd., Nanjing 210002, China).

## 3 Results

A previously healthy 5-year-old girl presented to our hospital with a 1-week history of involuntary movements, unsteady gait, and a progressively irritable temper. Initially, twitching of her mouth, writhing trunk, and swinging limbs were uncontrolled, but her symptoms progressed to gait imbalance and frequent tripping.

Upon admission, the patient appeared restless. Her consciousness was clear and she had no other neurological symptoms, splenomegaly, hepatomegaly, lymph node swelling, or fever. Physical examination revealed dysmetria and intention tremors on finger-to-nose testing. The patient was unable to stand on both feet simultaneously druing Romberg test. Alternating movement and heel-to-shin tests was unnable to cooperate. The patient also exhibited a slight decrease in muscle strength and tone. No abnormality of the cranial nerves was found. The deep tendon reflexes were elicited symmetrically. Pathological reflex examination was negative. The family history was unremarkable.

**FIGURE 1:**
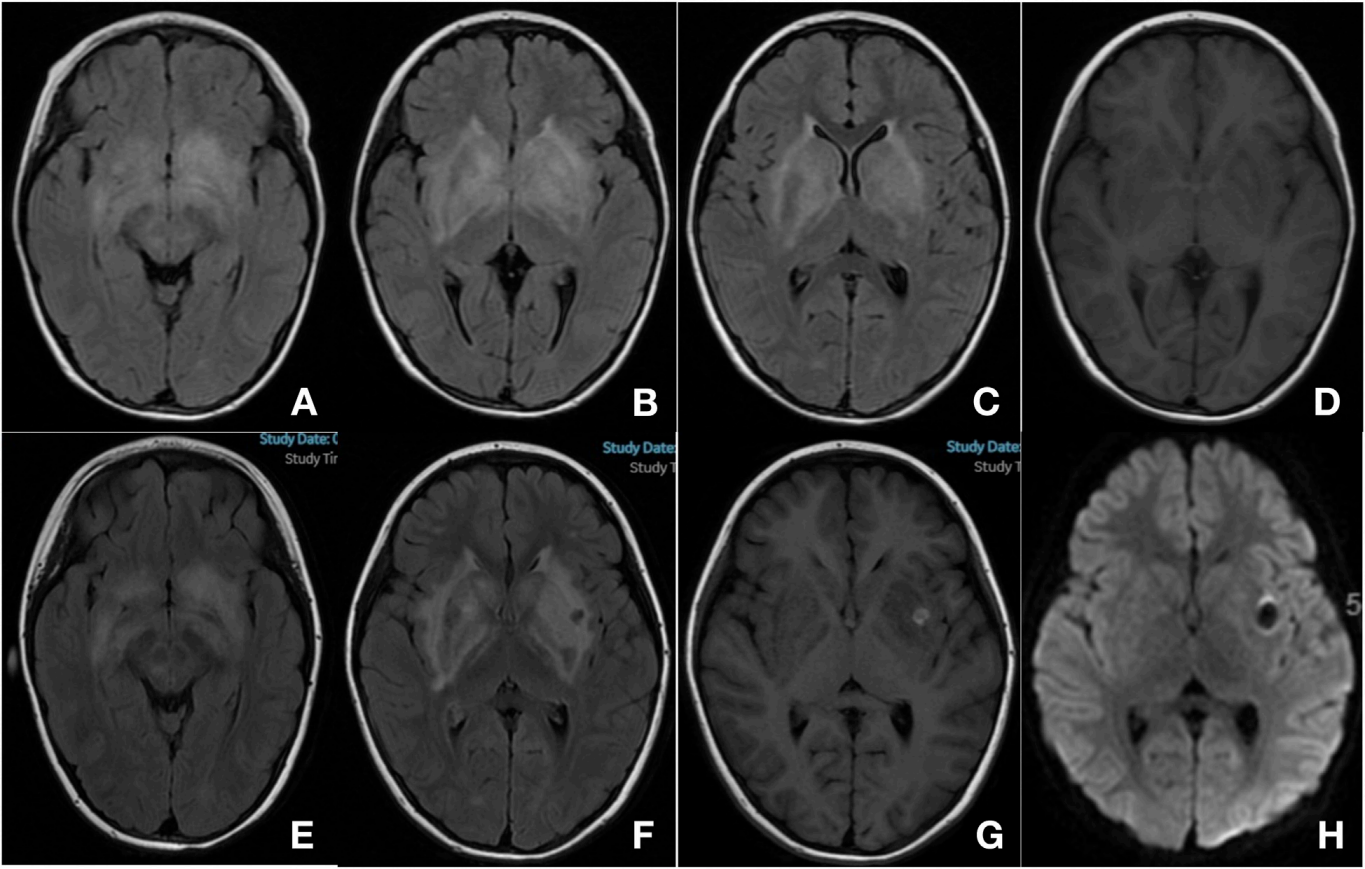
Cranial magnetic resonance imaging (MRI). MRI at 1 week of onset **(A–D)** bilateral basal ganglia, corona radiata, brainstem and anterior-posterior horns of lateral ventricle showed increased signal on T2-FLAIR **(A–C)** and decreased signal on T1 image **(D)** MRI at 1 month of onset **(E–H)** showed progression of the previously described lesions, and new-onset hemorrhage in the left basal ganglia [**(E–F)** T2-FLAIR; G: T1; H: DWI].

Laboratory test results showed that red and white blood cell counts, and platelet counts were within normal ranges (Table 1). Blood chemistry analyses, including liver enzymes, renal function, and electrolytes, were normal. Triglycerides increased slightly (Table 1). The erythrocyte sedimentation rate, ferritin level, coagulation function, thyroid function, and antinuclear antibody levels were also normal (Table 1). Cranial MRI showed FLAIR hyperintensities in bilateral white matter, basal ganglia, corona radiata, brainstem, and anteroposterior horns of the lateral ventricle (Figures 1A–D). Cervical, thoracic, and lumbar MRI revealed no abnormalities. Video EEG showed a normal occipital background rhythm without epileptic discharges. CSF examination revealed pleocytosis (9 cells/mL) with normal glucose, protein and immunoglobulin levels. The CSF IL-6 and IL-8 levels were also elevated. The CSF bacterial smears and pathogenic sequencing results were negative. Comprehensive autoimmune encephalitis-associated antibodies in serum and CSF were normal. Blood amino acid and urine organic acid screening results were negative.

**TABLE 1:** Summary of Lab results.

Test	Value	Reference	Interpretation
WBC	6.16 × 10^9^	4.40–11.90	Normal
Hb	126 g/L	112–149 g/L	Normal
RBC	4.8 × 10^12^	4.00–5.50 × 10^12^	Normal
Hematocrit	39.7%	34.0%–43.0%	Normal
PLT	289 × 10^9^	188–472 × 10^9^	Normal
Ferritin	25.45 ng/mL	13–150 ng/mL	Normal
Triglycerides	1.87 mmol/L	0.0–1.7 mmol/L	High
Fibrinogen	2.17 g/L	2.0–4.0 g/L	Normal
ALT	15.2 U/L	7–30 U/L	Normal
AST	27.3 U/L	14–44 U/L	Normal
CRP	0.02 mg/L	0–5 mg/L	Normal
ESR	2.0 mm/1 h	0–20 mm/1 h	Normal
soluble CD25 in CSF	69 pg/mL (CSF)	<6,400 pg/mL	Normal
soluble CD25 in blood	2,341 pg/mL (Blood)	<6,400 pg/mL	Normal
NK cell activity	17.9%	≥15.11%	Normal
ΔGranzyme B in NK	94.88%	≥77%	Normal
ΔGranzyme B in CTL	97.72%	≥6%	Normal
ΔCD107a in NK	10.29%	>10%	Normal
ΔCD107a in CTL (ΔMFI)	7.7	≥2.8	Normal
ΔPerforin (%) in NK	19.62%	≥81%	Low
ΔPerforin (%) in CTL	Below the detection limit	≥2%	Low

CTL, cytotoxic T lymphocyte; MFI, mean fluorescence intensity.

A diagnosis of an inflammatory disease of the central nervous system was presumed. Treatment with intravenous immunoglobulin (IVIG, 2 g/kg total) and intravenous methylprednisolone (IVMP, 4 mg/kg/d) resulted in clinical improvement. The patient’s involuntary movements were significantly reduced, and she could walk steadily, although she remained irritable. However, another brain MRI scan showed progression of the previously described lesions and a new-onset hemorrhage in the left basal ganglia (Figures 1E–H). A few days later, the result of WES returned and identified that she had a homozygous missense mutation in the *PRF1* gene (NM_001083116.3): c.1349C > T (p.Thr450Met). The variant c.1349C > T was classified as pathogenic accoding to American College of Medical Genetics and Genomics (ACMG) guidelines for variant classification (PVS + PM + PP), and has been previously reported as pathogenic variant associated with familial HLH (PMIDs: 39434014, 26903364, 25297583, 25233452, etc.). Parental sample testing revealed that her mother was heterozygous and her father was wild-type. Furthermore, we selected multiple short tandem repeat (STR) sequences and used STR polymorphic linkage analysis to confirm her consanguinity with her parents and identify the maternal uniparental disomy of the patient.

Further workup of bone marrow aspiration revealed the phenomenon of cell phagocytosis. Repeat CSF examinations revealed no pleocytosis, oligoclonal bands, or intrathecal IgG synthesis. Perforin expression testing revealed that perforin is essentially absent (Table 1). NK cell activity, Granzyme B, and cd107a mobilization were normal (Table 1). The blood and CSF-soluble CD25 levels were normal (Table 1). Considering the homozygous pathogenic variant of *PRF1* and supportive phenotype of the patient, a diagnosis of CNS-HLH was confirmed. The patient was transferred to the hematology department for chemotherapy. She was also a candidate for allogeneic bone marrow transplantation and HLA typing, and a search for a compatible stem cell donor was performed.

## 4 Discussion

HLH is a life-threatening hyperinflammatory syndrome that causes systemic inflammation and can lead to multiorgan failure and death (Griffin et al., 2020). It is characterized by edema, hepatosplenomegaly, and liver dysfunction (Griffin et al., 2020). Laboratory studies may show pancytopenia, coagulation abnormalities, hypofibrinogenemia, and hypertriglyceridemia (Griffin et al., 2020; Morimoto et al., 2016). The incidence of CNS involvement in HLH ranges from 10% to 73% (Solomon et al., 2018; Goo and Weon, 2007). HLH rarely presents with signs and symptoms that are isolated from the CNS (Benson et al., 2019). Patients with CNS-HLH are characterized by chronic inflammation restricted to the CNS that cannot be attributed to any other neuroinflammatory etiology, with no signs of systemic inflammation (Gupta et al., 2023; Benson et al., 2019). Here, we report the case of a 5-year-old girl diagnosed with CNS-HLH. Our case was unusual because the clinical presentation was restricted to the CNS and was alleviated by IVIG and steroid treatment. Uniparental disomy of the *PRF1* mutation confirmed the diagnosis.

Common neurological symptoms of HLH include seizures, irritability, encephalopathy, gait ataxia, headache, hypotonia, cranial nerve palsy, and meningismus (Malik et al., 2021; Lehrer et al., 2021). Our patient presented with involuntary movement and an unsteady gait as the main manifestations, which are uncommon in CNS-HLH. Multifocal bilateral white matter abnormalities, mainly in basal ganglia and corona radiata, were seen in our case. The neuroradiologic features of CNS-HLH were heterogeneous (Benson et al., 2019; Li et al., 2019; Blincoe et al., 2020; Debinski et al., 2021). Nonspecific diffuse or multifocal white matter abnormalities were most commonly observed. The cerebellum was frequently involved, most often manifesting as white matter change, with lesions also observed anywhere in the brain (Benson et al., 2019; Li et al., 2019). MRI changes are more often bilateral in CNS-HLH than unilateral in autoimmune demyelinating disease (Lehrer et al., 2021). Hemorrhage was also observed; however, this was rare. Spinal cord involvement has also been reported but appears to be even rarer than hemorrhage (Li et al., 2019).

HLH is related to several genes, including *PRF1, UNC13D, STX11, STXBP2, Rab27a, LYST, SH2D1A, BIRC4, ITK, AP3β1, MAGT1,* and *CD27*. Mutations in *PRF1*, which affect perforin expression in T and NK cells, are most common in CNS-HLH (Benson et al., 2019; Blincoe et al., 2020). Our patient harbored a homozygous missense mutation, c.1349C > T (p.Thr450Met), of *PRF1* gene, which was classified as pathogenic variant associated with familial HLH (Barmettler et al., 2016; Sun et al., 2014; Wang et al., 2014) and has been previously described in patients with CNS-HLH (Borda et al., 2024; Feng et al., 2020). For instance, Borda et al. (Borda et al., 2024) reported a 15-year-old male who was diagnosed as CNS-HLH and harbored compound heterozygous mutations of Thr450Met and Pro187Ser in *PRF1* gene. Feng et al. (Feng et al., 2020) reported four Chinese pediatric patients who presented with neurologic manifestations as initial clinical presentation of HLH due to *PRF1* mutation, and a heterozygous missense mutation Thr450Met that was present in two patients. In our patient, the absence of perforin expression in the blood also verified its pathogenicity. Maternal uniparental disomy is extremely rare, which indicates that suspicious genetic variants should not be dismissed. Timely recognition might be lifesaving, as hematopoietic stem cell transplantation might be well tolerated and effective for CNS-HLH (Gupta et al., 2023).

## 5 Conclusion

In conclusion, we report the case of a 5-year-old girl who presented with involuntary movements and an unsteady gait and was diagnosed with CNS-HLH due to uniparental disomy of a *PRF1* mutation. Owing to its similarities with other CNS diseases, CNS-HLH remains difficult to diagnose. Exome sequencing should be considered in patients with chronic or recurrent brain inflammation who show partial or no response to conventional treatment regimens.

## Data Availability

The datasets presented in this article are not readily available because of ethical and privacy restrictions. Requests to access the datasets should be directed to the corresponding author.
